# An ex vivo injury model perfused with whole blood reveals rapid mechanical and transcriptional changes within the spinal cord

**DOI:** 10.1002/btm2.70139

**Published:** 2026-03-23

**Authors:** Laura De Marchi, Hoi Yan Yu, Gianna Riviello, Sophia Orbach, Peter A. Galie

**Affiliations:** ^1^ Department of Biomedical Engineering Rowan University Glassboro New Jersey USA

**Keywords:** blood‐spinal cord barrier, ex vivo model, spinal cord injury, transcriptomics

## Abstract

Secondary spinal cord injury (SCI), which includes a cascade of both biophysical and biochemical processes that mitigate neurological recovery, is thought to initiate within minutes of the direct, primary injury. However, previous in vitro and in vivo‐based approaches are incapable of determining how quickly and to what extent these processes occur following injury. In contrast, the perfused bovine ex vivo indentation injury model described here represents a new platform to investigate the short‐term effects of observed phenomena like altered blood flow and blood‐spinal cord barrier disruption following traumatic SCI. The model can assess tissue changes on the scale of minutes to determine how rapidly the spinal cord responds to injury. Indentation tests simultaneously simulate a crush injury and measure the mechanical properties of the tissue using Hertz contact theory. Our results indicate that injured cords exhibit changes to bulk perfusion, as evidenced by laser speckle contrast imaging, in addition to decreased barrier function minutes after injury. Mechanical testing of the cord reveals that the tissue softening observed previously in the days following injury initiates as early as 30 minutes. Moreover, perfusing red blood cells resuspended in saline instead of whole blood mitigates the drop in elastic modulus, highlighting the importance of leukocytes and plasma proteins in mediating the extracellular matrix mechanical response after SCI. Transcriptomic analysis reveals key differences in gene expression within 15 minutes of injury, including those related to inflammation and the immune response. Identifying the timeframe and extent to which biomechanical and biochemical changes occur after injury provides a more complete understanding of secondary injury.

## Graphical abstract

A perfused ex vivo model of spinal cord injury shows that the tissue responds in as little as 15 minutes following a moderate injury.

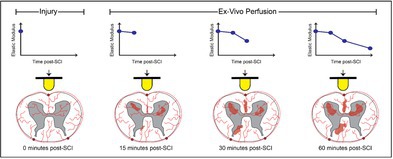

**Abbreviations**
ANOVA: Analysis of Variance
DAPI: 4',6‐diamidino‐2‐phenylindole
FITC: fluorescein isothiocyanate
GAP43: Growth associated protein 43
HSD: honestly significant difference
IACUC: Institutional Animal Care and Use Committee
NSF: National Science Foundation
RBFOX3: RNA Binding Fox‐1 Homolog 3
ROI: region of interest
TNFalpha: Tissue necrosis factor alpha
ZO‐1: Zonula occludens‐1

**Translational Impact Statements**

Our study introduces a perfused ex vivo spinal cord injury (SCI) model that reveals rapid mechanical and transcriptional changes within minutes of trauma. By replicating blood flow and barrier disruption, this model provides new insights into how inflammation and extracellular matrix remodeling contribute to secondary injury. These findings highlight the critical early window for intervention and suggest potential therapeutic targets to preserve spinal cord integrity. This model offers a valuable platform for testing treatments that may mitigate damage and improve recovery following SCI.

## 1 INTRODUCTION

The primary phase of spinal cord injury (SCI) involves both neuronal cell death and hemorrhage, processes known to alter levels of damage‐associate molecular patterns (DAMPs) in the central nervous system.^1^, ^2^ The ensuing inflammatory response is exacerbated by other aspects of secondary injury, a term first coined by Alfred Allen in 1911,^3^ which include breakdown of the blood‐spinal cord barrier (BSCB) responsible for limiting transport of cells and cytokines from the blood into the parenchyma.^4^ The timeframe that secondary injury processes initiate biophysical and biochemical changes in the tissue is currently unknown. For example, although previous studies have shown that transcriptional changes occur as quickly as 1 hour post‐injury,^5^ the shortest interval assessing altered mechanical properties of the cord after injury is 1 day.^6^, ^7^ Clarifying the dynamics of secondary injury and its effects on both tissue and cellular‐level properties improves our understanding of SCI and also has relevance for transplantable, regenerative scaffolds that seek to match the mechanical properties of the spinal cord.^8^, ^9^

Ex vivo models provide a platform to assess tissue properties in nearly real‐time due to the increased access and augmented control over the environment. Previously, organotypic slice culture (OSC) models have been used to study the biochemical post‐traumatic events of SCI^10^, ^11^, ^12^ as well as to evaluate the cell response to potential therapeutic strategies.^13^, ^14^ OSC models conserve both parenchymal and vascular tissue complexity of the tissue while maintaining significant viability of the cells up to 14 days after insult.^11^, ^15^, ^16^, ^17^, ^18^ However, although the controlled environment makes current ex vivo models a useful platform, their inability to perfuse blood flow through the tissue vasculature is a major limitation given the importance of BSCB breakdown in secondary injury. Changes to both bulk blood flow and microvascular barrier integrity cannot be modeled in OSC platforms without the addition of whole blood flow.

In vivo methods have also been developed to assess changes in spinal cord blood flow in the aftermath of SCI. Laser speckle contrast imaging relies on refracted scattered light to detect changes in superficial vasculature, and it has recently been used to monitor changes in perfusion levels after SCI.^19^, ^20^ Moreover, substantial advances in the capability of ultrasound to visualize blood flow in the injured spinal cord have been demonstrated in recent studies.^21^, ^22^ These studies indicate that local areas of the spinal cord undergo a substantial decrease in whole blood perfusion in the acute phase of injury. Although these methods provide insight into the rapid changes in blood flow caused by SCI, they are incapable of measuring changes in tissue properties in the immediate aftermath of the injury.

The perfusable ex vivo SCI model described here overcomes the limitations of both in vivo models and previous ex vivo OSC models. Perfusing the cord with whole blood facilitates assessment of the effects of BSCB breakdown, and the accessibility of the model allows for evaluation of the tissue and cellular properties within minutes of the injury. Both indentation experiments and bulk RNA sequencing are used to evaluate how a crush injury affects bulk mechanical properties and transcriptomics. This approach provides new insight into the mechanisms and dynamics of the processes associated with secondary injury.

## 2 MATERIALS AND METHODS

### 2.1 Solution preparation for spinal cord isolation and testing

Dissecting artificial cerebral spinal fluid (aCSF) was prepared using a previous protocol to maintain spinal cord tissue viability.^8^, ^23^, ^24^ The solution includes 191 mM sucrose, 0.75 mM K‐gluconate, 1.25 mM KH_2_PO_4_, 26 mM NaHCO_3_, 4 mM MgSO_4_, 1 mM CaCl_2_, 20 mM glucose, 2 mM kynurenic acid, 1 mM (+)‐sodium L‐ascorbate, 5 mM ethyl pyruvate, 3 mM myo‐inositol, and 2 mM NaOH. All the reagents were purchased from VWR, and solutions were prepared the day prior to spinal cord isolation and kept at 4°C.

### 2.2 Spinal cord and blood preparation

Bovine spinal cords were extracted from slaughtered steer at Bringhurst Meats (Berlin, NJ) and immediately submerged into ice‐cold aCSF within minutes of death. Simultaneously, bovine blood was collected in a quart‐sized container at Bringhurst Meats (Berlin, NJ) with 10 mL of a 10 mM sodium citrate solution and maintained on ice until the time of perfusion to prevent coagulation. Rowan University does not require IACUC approval for discarded tissue obtained from animals not euthanized specifically for the study. At the time of the experiment, the sections of the cord that were damaged during the extraction procedure were discarded and the remaining ones were divided into 10 cm sections. For experiments involving perfusion of resuspended red blood cells (rRBC), a 50 mL conical tube of whole blood was centrifuged at 1000 × *g* for 10 min, the plasma and buffy coat were removed, and the volume was replaced with 1× phosphate buffered saline (PBS) to maintain the original hematocrit. In experiments requiring Evans blue, the reagent (purchased from VWR) was added to blood at 1 wt.% prior to perfusion through the spinal cord.

All spinal cord tissue and blood samples were obtained from slaughtered steers. Therefore, all samples used in this study were male. As sex is a biological variable that can influence the response to SCI,^25^ the findings of this study may not fully generalize to female animals. Future studies incorporating both sexes are needed to determine whether sex‐specific differences influence the observed outcomes.

### 2.3 Indentation injury model and mechanical characterization

All the experiments were performed at room temperature within 6 hours of extraction. The dura mater was carefully removed from an approximately 10‐cm long spinal cord section to access the vasculature surrounding the pia mater. A spherical indenter with a 5‐mm radius was attached to the top plate of a Shimadzu mechanical tester, and a compressive force was applied to the anterior side of the spinal cord section at least 5 cm away from its edges at a deformation rate of 6 mm/s for a 6‐mm indentation. Figure S1 provides a photograph of a typical spinal cord section prior to indentation.

The indentation exerted by the axial testing system was used to both impart a crush injury on the cord and collect indentation data simultaneously. Each cord section was tested in pairwise fashion: an initial indentation was performed to both measure baseline mechanical properties and initiate a crush injury. Immediately after the injury, the anterior spinal cord artery (ASA) was cannulated with a 25‐gauge needle connected to an Arduino‐controlled peristaltic pump to perfuse whole blood containing sodium citrate to prevent coagulation during the experiment or reconstituted red blood cells (rRBC). The peristaltic pump was operated at a constant speed of 30 rpm, which yielded a blood flow rate of approximately 1 mL/min prior to cannulation. Then, indentation with the mechanical tester was performed to a depth of 6 mm after perfusing for either 15, 30, or 60 minutes. All measurements were terminal so that each timepoint measured a unique segment. The percent change in elastic moduli between the time of injury and at varying time points was calculated by applying the Hertz model to the impact force measurements collected by the mechanical tester.

### 2.4 Laser speckle contrast imaging

A portable laser speckle contrast imaging (LSCI) device (RFLSI‐ZW, RWD Life Science) with a near‐infrared laser diode at 785 nm was mounted on the bench countertop with an extendable arm and used to monitor blood flow for up to 60 min after indentation injury. The indentation was applied using the setup shown in Figure S1 and then transferred to the imager. The imager containing the laser diode and the camera was positioned perpendicular to the area of injury about 16‐cm above the spinal cord section and the focus was adjusted to image an area of about 3.82 × 2.86 cm^2^. Images were taken at a frame rate of 15 fps with an exposure time of 20‐ms for 5 s and analyzed through the device software to collect blood perfusion units (BPU) averages and standard deviation values for the region of interests selected. The quantification of the LSCI images was performed by normalizing the BPU mean over the injury region of interest to the BPU mean over the control region of interest, which was selected in a portion of the cord removed from the area of indentation. At least six measurements for condition were obtained and subjected to linear regression analysis.

### 2.5 Bovine spinal cord immunohistochemistry

Bovine spinal cord sections were washed in PBS and fixed in 4% paraformaldehyde for 30 min. After fixation, the sections were embedded in OCT compound and cryopreserved at −80°C. Each section was cut into 20‐μm slices with a cryostat and mounted on microscope slides. The slides were washed with PBS with Triton X‐100 (PBST) and blocked with 1% normal donkey serum (Sigma‐Aldrich) for 1 hour at room temperature. The slides were incubated for 24 hours in a solution consisting of 0.1% normal donkey serum and a mouse monoclonal anti‐CD68 primary antibody (AB_2538148, Life Technologies) diluted at a ratio of 1:100. The slides were washed 3× in PBS and a goat anti‐mouse secondary antibody conjugated to AlexaFluor 488 (Life Technologies) was added at a ratio of 1:100 and incubated at room temperature for 2 hours. After thoroughly washing the slides with PBS, they were mounted in anti‐fading mounting media containing DAPI stain (VWR) and stored at 4°C.

### 2.6 Confocal microscopy and quantification

Immunohistological slides of the bovine spinal cord sections were imaged on a Nikon A1 laser scanning confocal microscope. Images were processed and quantified in ImageJ and Excel. The Evans blue ratio was quantified by measuring the ratio of intensity within the lumen and in the extravascular space (Figure S2). At least three measurements were made in distinct vasculature to calculate an average and standard deviation for the ratio between lumen and extravascular intensity.

### 2.7 Bulk RNA sequencing

Spinal cord segments from a total of six animals were flash frozen in liquid nitrogen after 15 min of whole blood perfusion for both injured and control conditions. Prior to freezing, samples were perfused with PBS to minimize interference from blood immune cells. The frozen tissue samples were solubilized in TRIzol reagent to separate RNA from DNA and protein. The RNA was further purified using an RNEasy spin column prior to shipping to GeneWiz for bulk RNA sequencing. GeneWiz completed quality control of the samples, rRNA depletion, and Next Generation RNA sequencing before returning FASTQ files for analysis. The FASTQ files were aligned to the ARS‐UCD2 bovine reference genome and quantified using the Rsubread package in R.^26^ DESeq2 was used to normalize the gene‐count matrix, filter low reads, and conduct differential expression.^27^ To complete pathway analysis, bovine genes were first converted to their human orthologs using biomaRt.^28^ Enriched biological pathways were identified with Gene Set Enrichment Analysis.^29^ Gene sets were selected from the hallmark (H), curated (C2), and gene ontology (C5) collections from the Molecular Signatures Database.

### 2.8 Statistics

The mechanical testing experiments were performed in triplicate on sections derived from 16 distinct ex vivo spinal cords. The LSCI was performed on two separate sections of the same cord (one control and one injured section) over a 1‐h time frame with *n* > 6 measurements for each sample. Immunohistochemical staining was performed on slides from the same section of the spinal cord but each sample was from a different animal (*n* = 5). Two‐sample *t* tests, one way ANOVA, and post hoc Tukey's HSD tests were used to calculate the statistical significance of our results. Statistical significance between different perfusion conditions at the same time points was calculated using two sample *t* tests, assuming normal conditions and unequal variances. To determine whether rRBC perfusion resulted in a percentage decrease greater than zero, multiple one‐sample *t* tests with Bonferroni adjustments were completed comparing the means to zero. All *p*‐values lower than 0.05, 0.01, 0.001, and 0.0001 were reported as *, **, ***, **** ﻿﻿respectively.

## 3 RESULTS

### 3.1 Ex vivo model validation

**FIGURE 1:**
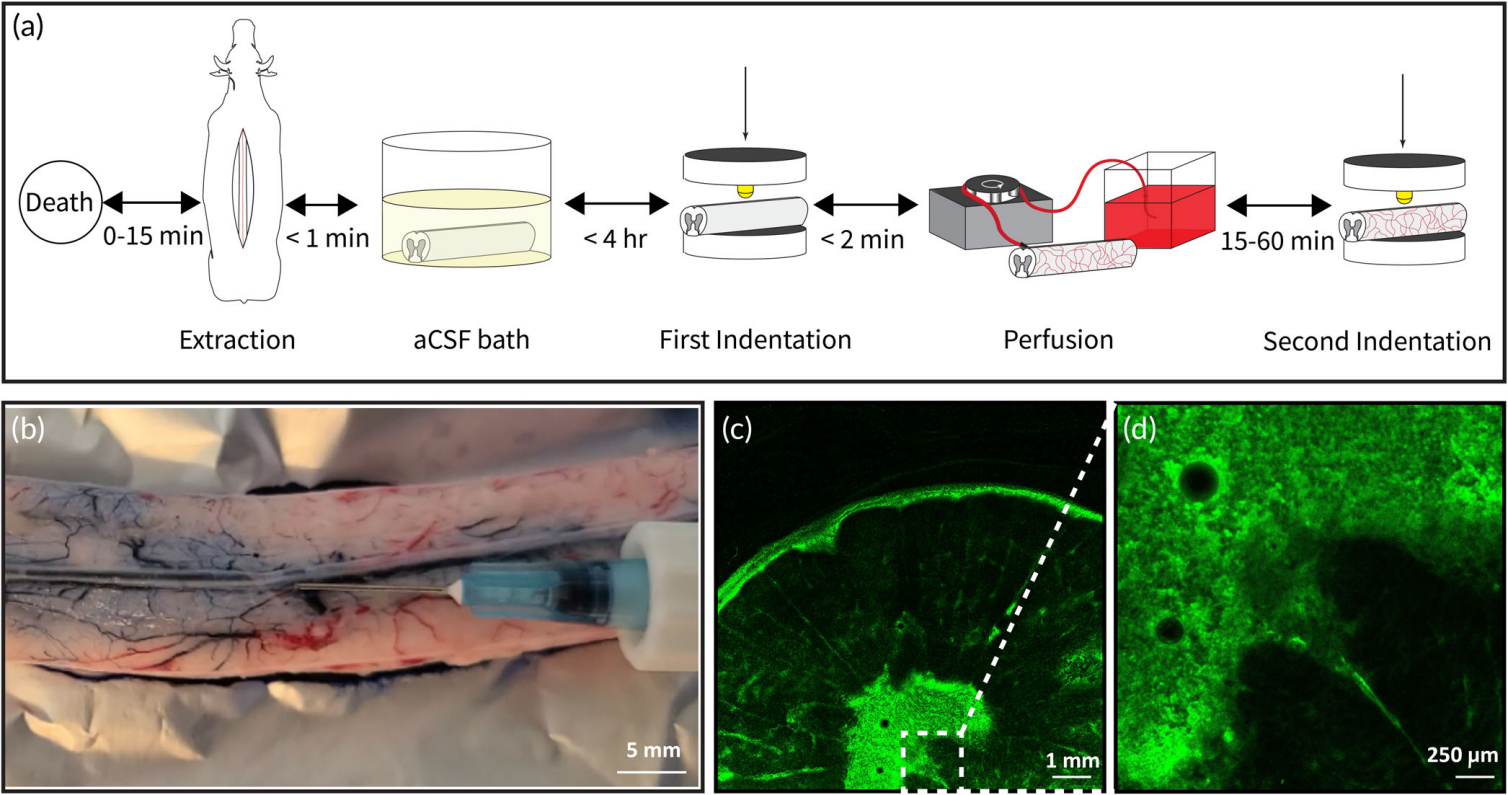
Perfused ex vivo injury model schematic and validation. (a) Schematic of compression model. (b) Perfusion of Evans blue and (c, d) coronal sections following perfusion with 2 MDa‐FITC dextran. Scale bar = 1 mm in (c) and 250 μm in (d). aCSF, artificial cerebral spinal fluid.

The primary advantage of the ex vivo model is its ability to perfuse whole blood through the vasculature of the spinal cord, but experiments were necessary to assure that cannulating the anterior spinal artery resulted in parenchymal perfusion within the spinal cord segments. Figure 1a shows an overall workflow of the ex vivo experiments. Spinal cords were isolated directly into artificial cerebrospinal fluid, which has previously been used to preserve ex vivo tissue for mechanical testing as determined by staining for myelin at several time points.^8^ Pial vasculature perfusion was first verified by circulation of 1 wt% Evans blue‐bovine serum albumin solution (Figure 1b), and 2 MDa FITC‐labeled dextran in saline was then used to ensure successful perfusion through the parenchymal vascular bed. Coronal sections of the white and gray matter show the latter displays a higher density of vasculature following perfusion with the FITC‐dextran (Figure 1c). A higher magnification image of the gray matter demonstrates that the ex vivo approach is capable of perfusing not only penetrating arteries but also the microvasculature within the gray matter (Figure 1d).

### 3.2 Measuring pial vasculature perfusion following injury

**FIGURE 2:**
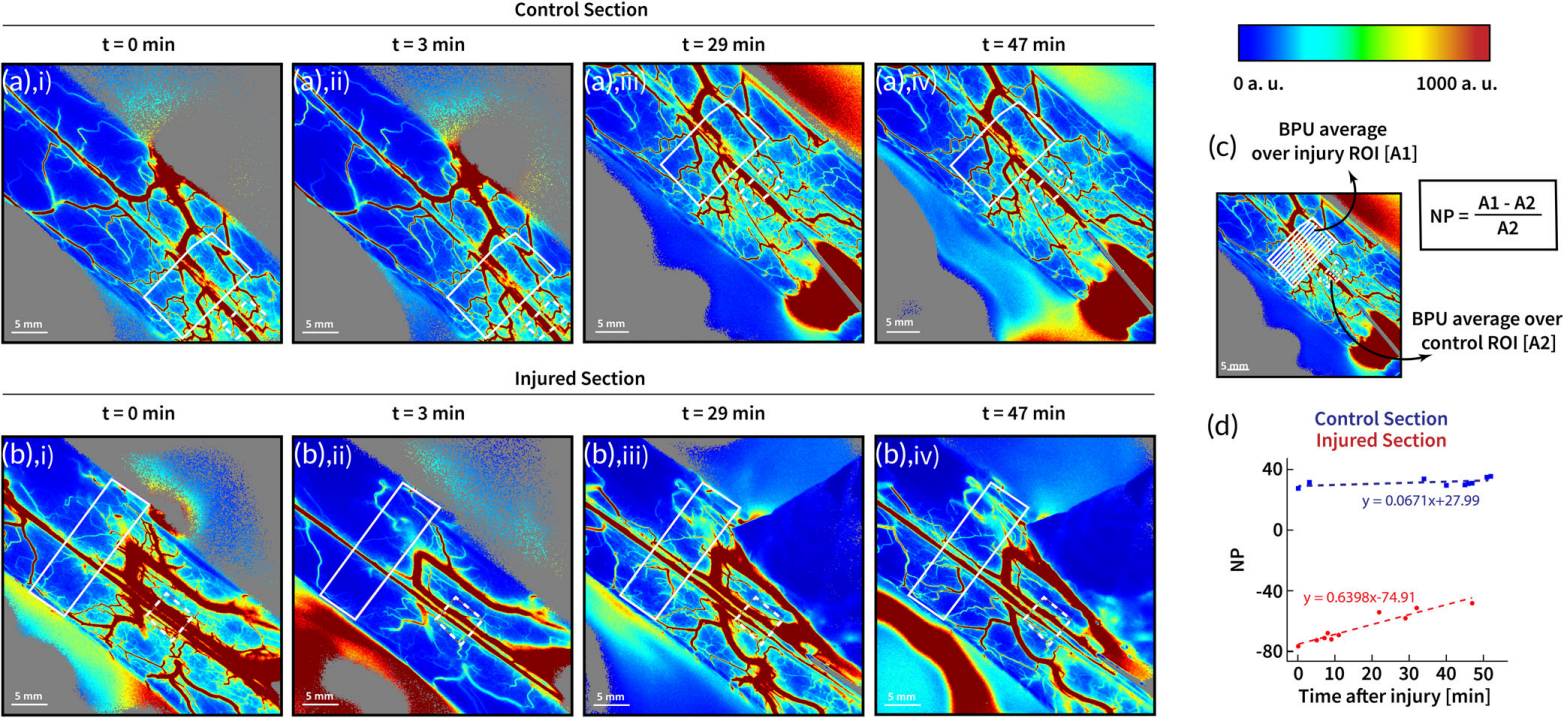
Laser speckle contrast images of bovine spinal cord perfused sections. Speckle contrast images of the perfused spinal cord sections for (a) control, (b) and injured section at (i) 0 min, (ii) 3 min, (iii) 29 min, and (iv) 47 min. (c) A schematic of the quantification of normalized flow. (d) Perfusion values over time for both samples with linear regression represented by dotted lines. Two separate sections of the same cord (one control and one injured section) were measured at least six times (*n* > 6) to compare differences in blood flow. BPU, blood perfusion units.

Having demonstrated the ability to perfuse the parenchyma of the tissue, LSCI was used to evaluate the effects of injury on blood flow in the pial vasculature. These experiments serve as an additional validation of the ex vivo model since previous studies have established that the spinal cord experiences hypoperfusion following injury.^30^ Figure 2a,b shows the flux heatmap images comparing the changes in superficial perfusion between an uninjured control section (Figure 2a,i–iv) and an injured section (Figure 2b,i–iv) at 0, 3, 29, and 47 minutes after injury. To quantify the changes in perfusion over time for each condition and account for the differences in topologies between the two sections of spinal cord, the ROI corresponding to the area of indentation was normalized to a reference area closer to the cannulation site that did not experience indentation (Figure 2c). This normalization accounted for changes in blood flow during the experiment. Quantification of the flux heatmap images (Figure 2d) and linear regression analysis of the BPU averages show a relatively constant level of perfusion through the control section (rate of perfusion increases at 0.0671/min), compared to the injured section (a rate of 0.6398/min), which was nearly an order of magnitude higher. These results suggest that the ex vivo model replicates the initial hypoperfusion phase in the spinal cord observed using in vivo ultrasound measurements and that the rate of perfusion increases as a function of time in the injured section.

### 3.3 Immunohistological evaluation of microvascular integrity

**FIGURE 3:**
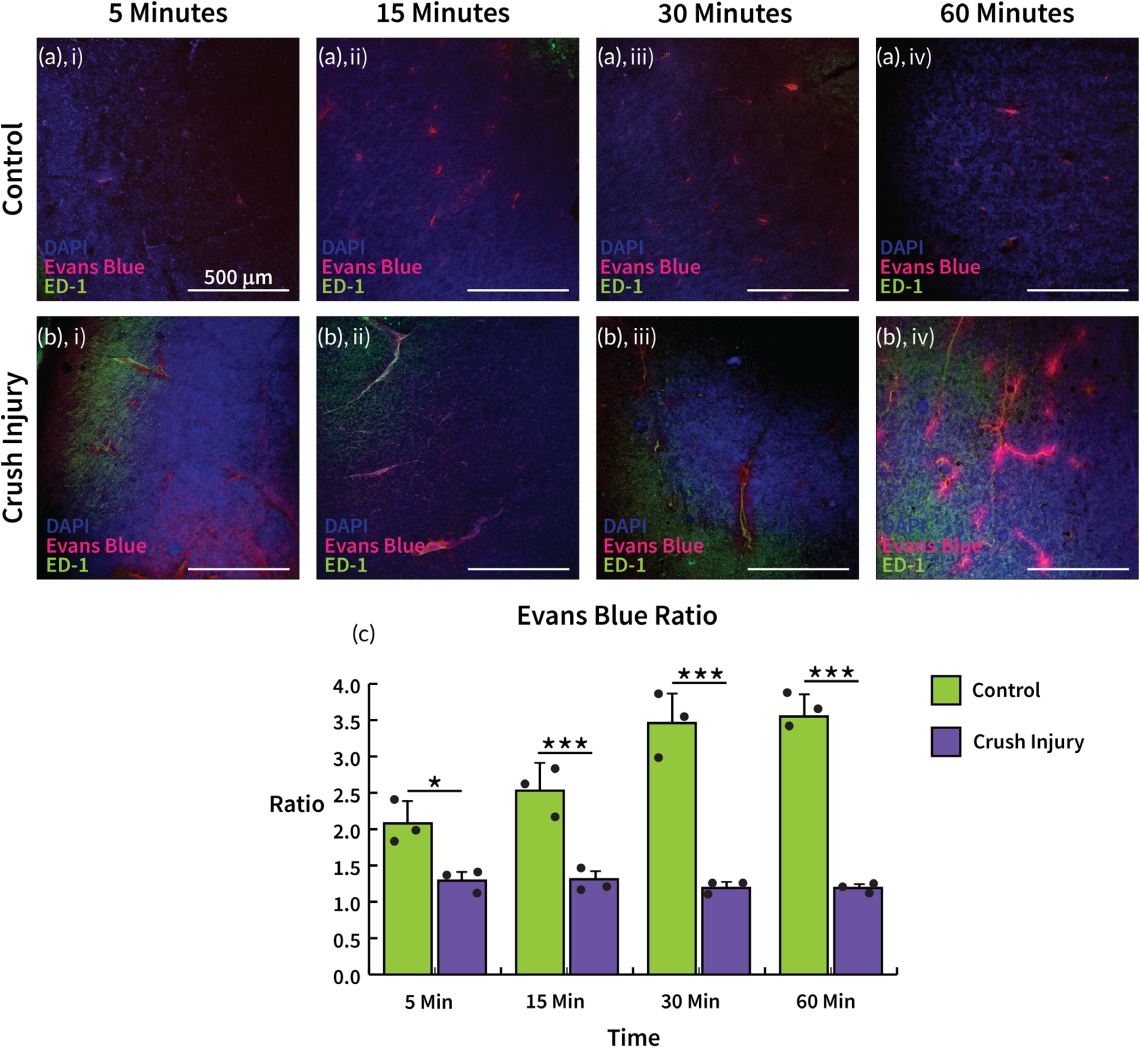
Immunohistochemical images of (a) control and (b) injured sections of spinal cord. Images shows DAPI (blue), Evans blue (red), and ED‐1 (green) at 5 min (a, i; b, i), at 15 min (a, ii; b,ii), at 30 min (a, iii; b, iii), at 60 min (a, iv; b, iv). (c) Quantification of Evans blue ratios (*n* = 5 per experimental condition). All *p*‐values lower than 0.05 and 0.001 were reported as *, *** ﻿﻿respectively.

An additional validation that the ex vivo model is representative of SCI necessitates histological evaluation of the integrity of the BSCB since breakdown of the barrier is an established characteristic of secondary injury. Evans blue was added to the whole blood perfusing the spinal cord segments after injury as well as control segments that did not undergo indentation. Figure 3 shows immunohistochemical images of control (Figure 3a,i–iv) and injured sections (Figure 3b,i–iv), which were used to quantify Evans blue extravasation at 0, 15, 30, and 60 minutes. The comparison of the ratio of Evans blue intensity in the lumen of vasculature and immediate extravascular space showed significant differences between injured and control cords. The ratio of Evans blue was significantly higher in the non‐injured controls for all four time points, evidence of BSCB breakdown in the injured samples. Moreover, an ANOVA revealed that time was a significant variable, indicating that the barrier function improved over the hour period of whole blood perfusion (Figure 3c). In contrast, post hoc Tukey tests found no significant differences between injured samples, suggesting that barrier disruption did not significantly change with time following injury. The increase in barrier function in response to the application of blood flow is consistent with previous studies indicating that fluid shear stress improves barrier function.^31^ Moreover, histological sections from the injured cord stained positive for ED‐1, a marker for microglia activation, providing evidence that BSCB breakdown correlated with an inflammatory response following injury.

### 3.4 Mechanical changes following injury

**FIGURE 4:**
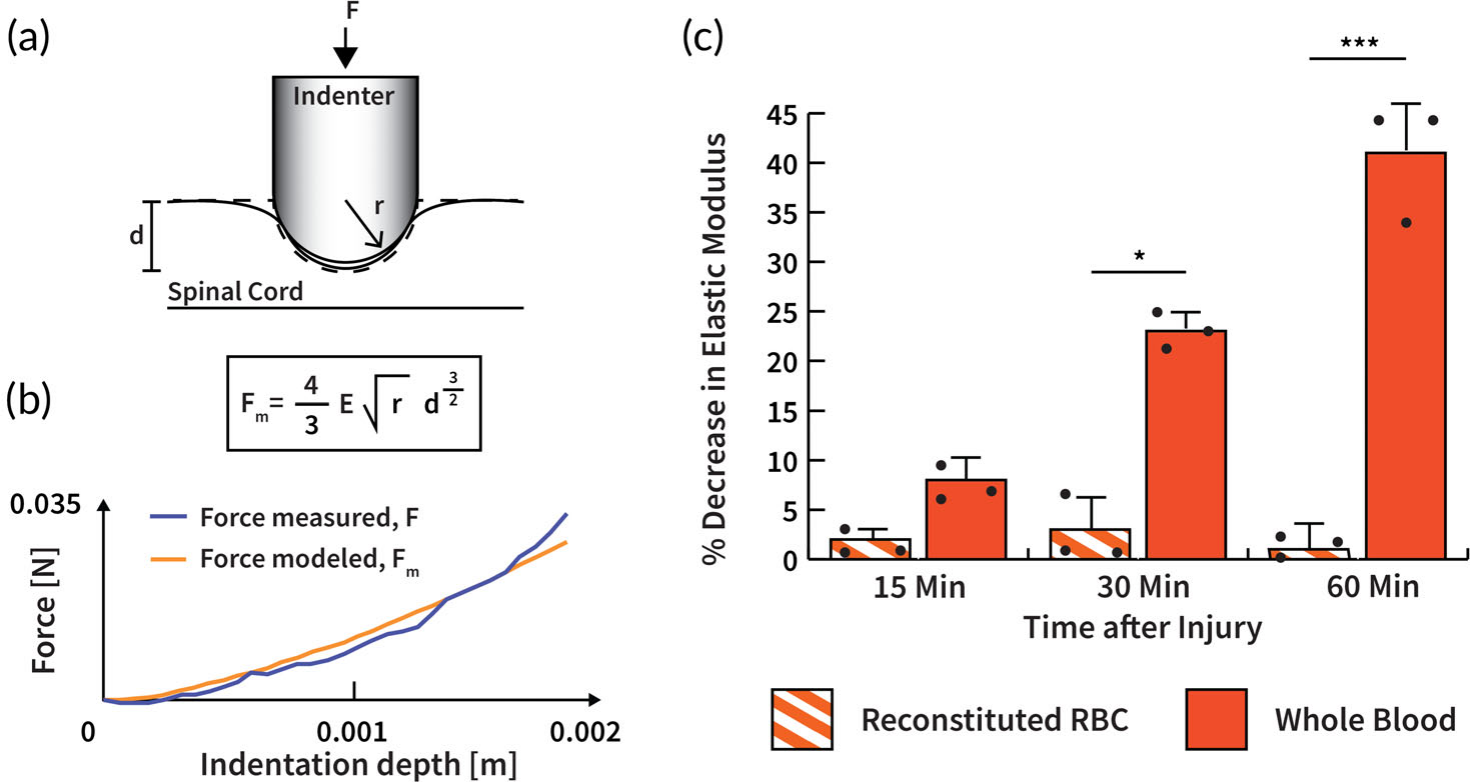
Mechanical testing analysis. (a) Indentation schematic. (b) Representative force‐indentation curve and fitting using the Hertz equation with a least squares fit. (c) Percentage decrease in mechanical properties from the time of injury to the second measurement in cords perfused with either whole blood or reconstituted red blood cells (rRBC) (*n* = 3 per experimental condition for each time point). All *p*‐values lower than 0.05 and 0.001 were reported as *, *** ﻿﻿respectively.

Having validated that the ex vivo model represented key aspects of SCI, experiments were conducted to determine the dynamics of changes to the mechanical properties of the cord. The mechanical testing system used to indent the cords provided the ability to simultaneously injure the cord and gather force‐deformation data that was used to derive its stiffness from the Hertz equation (Figure 4a,b). The elastic modulus of each cord segment was measured at the time of injury and then at either 15, 30, or 60 min later to determine the percent change (Figure 4c) from its initial value. Perfusion of the spinal cord with whole blood resulted in significant differences in elastic modulus decrease compared to the sections perfused with rRBC. After 30 min of perfusion, the elastic moduli for the sections perfused with whole blood decreased by 23.63% ± 1.31% compared to the 3.4% ± 2.74% of rRBC‐perfused sections. The elastic moduli decrease significantly increased over time and, at 60 min after injury, whole blood perfused sections showed a 41.44% ± 4.91% decrease compared to the 1.05% ± 1.98% decrease of sections perfused with rRBC (Figure 4c). Multiple one‐sample *t* tests with Bonferroni adjustment revealed that rRBC‐perfused sections did not exhibit a percent decrease greater than zero. Taken together, these results suggest that leukocytes and components of the plasma mediate the reduction in tissue stiffness that occurs within minutes of injury. Further evidence was provided by additional experiments performed in the absence of perfusion (at both room temperature and 37°C), which showed that the percent decrease in elastic modulus in the non‐perfused cords was significantly less than cords perfused with whole blood at both temperatures (Figure S3).

### 3.5 Transcriptional changes following injury

**FIGURE 5:**
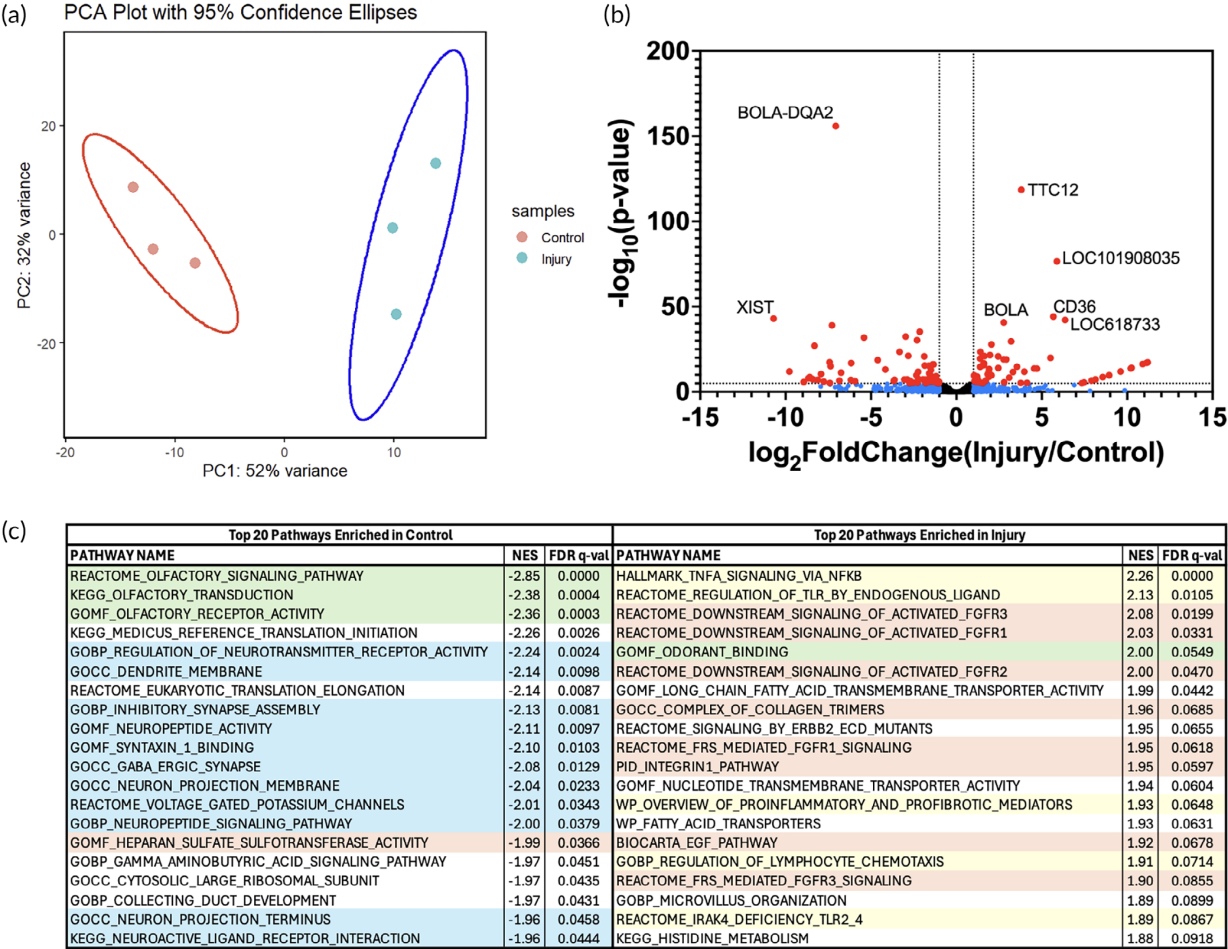
Unsupervised RNA sequencing results. (a) Principal component analysis (PCA) of non‐injured control and injured animals. (b) Volcano plot showing the most differentially expressed genes in injured cords. (c) Pathway enrichment analysis for both control and injured samples. Olfactory signaling (green), neural function (blue), inflammation (yellow), and extracellular matrix (ECM)‐related (orange) (*n* = 3 per experimental condition).

Previous studies have highlighted transcriptional changes that occur in the aftermath of a SCI, though at a timescale of several hours.^5^, ^32^ Therefore, cord samples were processed 15 min following injury and underwent bulk RNA sequencing to determine if transcriptional changes occur within a shorter time frame. The principal component analysis shown in Figure 5a indicates substantial differences in gene expression between the two groups of samples. The most differentially expressed genes are highlighted in the volcano plot in Figure 5b and include genes associated with inflammation and the immune response: the bovine major histocompatibility complex (BOLA) and CD36. Figure 5c provides the top 20 enriched pathways in both the control and injured samples. The most enriched pathways in the control samples involved olfactory signaling which includes genes for calmodulin 1 (CALM1) and calmodulin kinase 2 (CAMK2A and CAMK2B), which have previously been associated with differential expression following SCI.^32^ Multiple neural signaling pathways were also enriched in control samples, indicative of the effect of primary injury on damaging neurons. These findings were consistent with the expression of cell‐specific markers for astrocytes, microglia, neurons, and oligodendrocytes. GAP43 and RBFOX3 (NeuN) were significantly reduced in response to injury, suggesting neuronal loss or dysfunction following injury (Figure S4). In injured cord sections, several of the enriched pathways were associated with extracellular matrix (ECM)‐related signaling. Specifically, pathways related to fibroblast growth factor (FGF) were among the most enriched pathways. However, analysis of specific genes for the basement membrane (collagen 4 isoforms COL4A1, COL4A2, etc. and laminin chains LAMA1, LAMB1, LAMC1) and heparan sulfate proteoglycan (HSPG2) revealed no significant differences between control and injured samples (Table S1). Additionally, no significant differences in tight junction proteins, including ZO‐1 (adjusted *p*‐value = 0.504), claudin‐5 (adjusted *p*‐value = 0.332), and occludin (adjusted *p*‐value = 0.887) were observed, despite the increases in barrier permeability revealed by the extravasated Evans blue in the injured samples. These results suggest that transcription of these genes was not altered at the 15‐min time point. The most enriched pathway in the injured cords was related to TNFα signaling, which is consistent with an increased immune response that occurs following BSCB breakdown.

**FIGURE 6:**
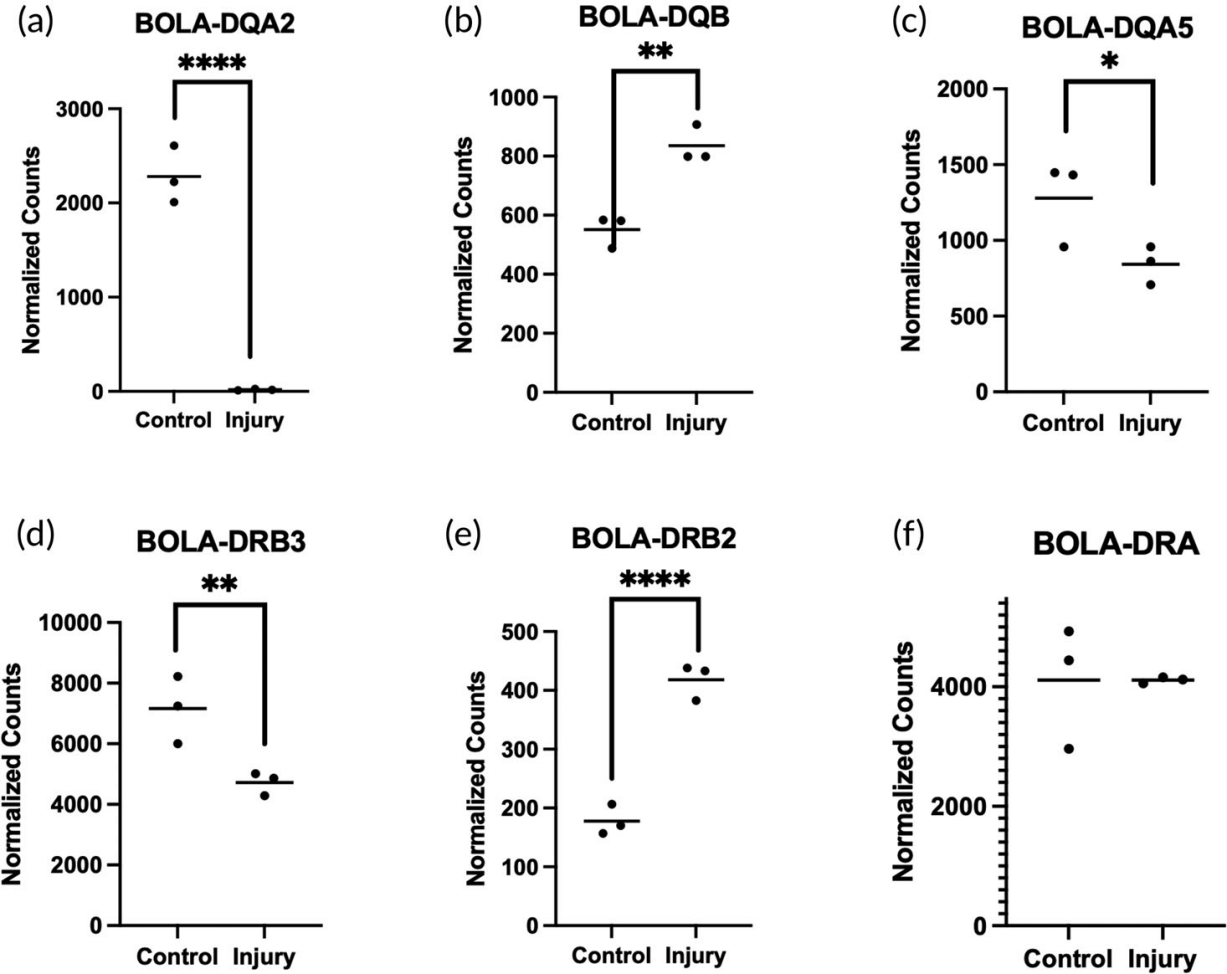
Differential expression of bovine major histocompatibility complex (BOLA) Class IIa genes including (a) DQA2, (b) DQB, (c) DQA5, (d) DRB3, (e) DRB2, (f) DRA (*n* = 3 per experimental condition). All *p*‐values lower than 0.05, 0.01, 0.001, and 0.0001 were reported as *, **, ***, **** respectively.

Parsing the differences in immune‐related gene expression revealed trends specifically within BOLA (bovine equivalent of major histocompatibility complex [MHC])‐associated genes. Genes associated with MHC Class I showed no significant differences between control and injury samples. In contrast, several genes in MHC Class II were significantly altered in the injured samples (either increased or decreased) (Figure 6). Different isoforms of MHCs are connected to different immune responses; thus, this variation is likely indicative of a heterogenous immune response. These connections are not well‐studied in bovine samples; thus, we cannot make a direct connection between the BOLA‐isoforms and the response of immune cells without further experimentation.^33^, ^34^, ^35^ Nonetheless, these results provide further evidence of an altered inflammatory environment in the injured cord only 15 min after the onset of injury, far sooner than observed in previous studies. In order to assess whether altered transcription persisted beyond 15 min, cords were processed at 60 min post‐injury. The volcano plot in Figure S5 indicates no significant differences in gene expression between injured cords at 15 and 60 min.

## 4 DISCUSSION

The perfused ex vivo model described in this study provides unprecedented insight into the rapid cellular and tissue‐level response to SCI. Transcriptional changes are observed as early as 15 min following injury, and there is a significant decrease in the elastic modulus within 30 min of cord segments perfused with whole blood. Taken together, the differential gene expression related to the immune response and the finding that perfusing rRBC mitigates tissue softening suggest that inflammation and ECM remodeling are closely related processes in the aftermath of SCI. This result supports previous studies that demonstrate the importance of leukocyte infiltration into the surrounding tissue in the promotion of ECM remodeling and its subsequent mechanical changes.^36^, ^37^ Further experiments are needed to investigate the specific mechanisms by which the extravasation of leukocytes, inflammatory cytokines, and other blood components may contribute to the reduction of mechanical properties observed following SCI. Nonetheless, this study both establishes the perfused ex vivo model as a reliable platform to interrogate acute injury and clarifies the rapid response at both transcriptional and matrix levels.

The results highlight the importance of including whole blood perfusion in the ex vivo SCI model. Changes to blood flow and reduced integrity of the BSCB are established phenomena in the aftermath of injury and therefore crucial to emulate in an injury model. Monitoring pial vasculature with LSCI indicates that the hypoperfusion observed in animal models^21^, ^22^, ^30^, ^38^ is recreated in the ex vivo model. The causes underlying spinal cord hypoperfusion are not clear, though previous studies have suggested that venous stasis and intrathecal hypertension may be contributing factors.^39^ However, the removal of the dura in these experiments appears to contradict the established importance of intrathecal hypertension for reducing blood flow. Future studies using the ex vivo model can investigate the role of pericyte contraction and other potential mechanisms underlying hypoperfusion. Additionally, the model also captures the breakdown of the BSCB, with Evans blue extravasation increasing with time after injury. The causes of barrier breakdown, specifically determining whether the reduced fluid shear stress caused by hypotension contributes to the reduced barrier integrity, can also be pursued in future work. In particular, a more detailed assessment of endothelial tight junctions, which are crucial for the function of the BSCB, can provide further insight into the results observed here. Although RNA sequencing showed that gene expression of tight junction proteins was not significantly altered at the early time points following injury, Evans blue extravasation in injured cords provides evidence of barrier disruption. Recent studies in our lab demonstrate that the proteomic profile of blood is altered by different hemodynamic factors^40^ and similar analysis of the blood perfused through the cord can be conducted. Additionally, future studies can also assess whether injury severity alters the dynamics of tissue softening and changes in gene expression. A previous study found that injury severity was dictated by indentation depths at the low strain rates used in these experiments.^41^ Scaling the 6‐mm indentation in the bovine spinal cord to the rat cords used in that study indicates that the ex vivo cord experienced a moderate injury that would be associated with substantial functional loss.

Clarifying the immune system response and the associated ECM remodeling immediately following injury is crucial to understanding the processes that govern secondary injury. Previous studies have identified inflammation and macrophage infiltration as crucial mediators of SCI.^42^, ^43^ In the brain, microglia undergo activation within minutes of an insult.^44^, ^45^ Our immunohistochemical results support these findings by showing that Evans blue diffuses outside of blood vessels as early as 5 min after injury and exhibits a significant progression as time increases. Moreover, positive staining of ED‐1, a macrophage/microglia marker, in the parenchyma of the tissue appears more prominent in injured samples compared to controls even at 5 min post‐injury. However, ED‐1 was not identified as a differentially expressed gene in the transcriptomic analysis. The cause of the discrepancy could be differences in gene expression and protein expression or residual blood within the spinal cord vasculature that remained in the samples during mRNA collection. Future studies will parse differences in transcription and translation by comparing these RNA sequencing results to proteomic analysis.

The bulk transcriptional results provide further insight into the importance of the inflammatory response in addition to the rapid nature of how expression of these genes is altered. The RNA sequencing results are consistent with previous evaluations of transcriptional changes in the acute phase of SCI and therefore provide new insight into the speed at which pathological changes occur after injury. For example, at 6 hours post‐injury, a significant mitigation in GAP43 expression was observed in a previous mouse model.^32^ The authors speculated that this reduction was indicative of neurite retraction. Therefore, the significant difference observed here suggests that this retraction is underway within 15 min of the injury. That study also showed a reduction in calmodulin and calmodulin kinase, the genes associated with the olfactory signaling pathway enriched in the control sample. Differential expression of MHC class II genes has also been recognized in previous animal models,^46^ though measured several weeks after injury. Identifying the rapid nature of these transcriptional changes is crucial to not only understand the pathology of SCI but also to inform the timing of potential treatments. For example, several approaches to activate FGF signaling have been pursued in animal models and clinical trials for SCI repair.^47^ In this study, several pathways associated with FGF were already enriched in the injury model and attempts to further simulate FGF signaling in the acute phase may exacerbate injury.

Future studies are needed to determine how the inflammatory signaling highlighted in this work influences the mechanical properties of the spinal cord following injury. While our findings suggest a link between immune activation and ECM remodeling, the specific contributions of cytokine signaling and matrix‐degrading enzymes to tissue softening remain unclear. By leveraging the perfused ex vivo model to systematically dissect these pathways, future research can clarify how inflammation‐driven ECM alterations contribute to secondary injury and identify potential strategies to preserve spinal cord integrity in the acute phase of trauma.

## 5 CONCLUSIONS

In summary, our perfused ex vivo SCI model provides a novel platform to investigate the immediate biomechanical and biochemical responses to trauma. By demonstrating that significant mechanical and transcriptional changes occur within minutes of injury, we highlight the critical early window in which secondary damage begins to unfold. The link between inflammation, ECM remodeling, and mechanical softening suggests potential therapeutic targets to mitigate tissue degradation. Future studies leveraging this model can refine our understanding of injury mechanisms and guide the development of early interventions to preserve spinal cord integrity and improve patient outcomes.

## AUTHOR CONTRIBUTIONS

LDM and PAG designed and performed experimental studies; HYY, GR, and SO provided computational analysis; LDM and PAG wrote the initial draft; SO and PAG oversaw document revisions.

## FUNDING INFORMATION

This work was supported by NSF 2233318 (Peter A. Galie).

## CONFLICT OF INTEREST STATEMENT

The authors have no conflict of interest to declare, and no artificial intelligence was used to process or present data or in the preparation of this manuscript.

## Supporting information


**Figure S1.** Photograph of a spinal cord section prior to indentation by the mechanical testing system.
**Figure S2.** Schematic showing the quantification method of (A) a representative IHC image for (B) Evans blue ratio.
**Figure S3.** Mechanical testing analysis comparing perfused versus non‐perfused samples. Percentage decrease in mechanical properties from the time of injury to the second measurement in cords.
**Figure S4.** Expression of cell type‐specific markers for (A) astrocytes, (B) microglia, (C) neurons, and (D) oligodendrocytes.
**Figure S5.** Volcano plot comparing differential gene expression between cords processed at 15 and 60 min post‐injury.

**Table S1.** Differential expression between injured and non‐injured controls.

## Data Availability

The authors have no conflicts of interest to declare, and the data that support the findings of this study are openly available in Harvard Dataverse at https://dataverse.harvard.edu/dataverse/galielab.

## References

[1] Zhang Y‐K , Liu J‐T , Peng Z‐W , et al. Different TLR4 expression and microglia/macrophage activation induced by hemorrhage in the rat spinal cord after compressive injury. J Neuroinflammation. 2013;10:1‐15. 24015844 10.1186/1742-2094-10-112 PMC3847110

[2] Kigerl KA , Lai W , Wallace LM , Yang H , Popovich PG . High mobility group box‐1 (HMGB1) is increased in injured mouse spinal cord and can elicit neurotoxic inflammation. Brain Behav Immun. 2018;72:22‐33. 29175543 10.1016/j.bbi.2017.11.018 PMC6681463

[3] Allen AR . Surgery of experimental lesion of spinal cord equivalent to crush injury of fracture dislocation of spinal column: a preliminary report. JAMA. 1911;57(11):878‐880.

[4] Figley SA , Khosravi R , Legasto JM , Tseng YF , Fehlings MG . Characterization of vascular disruption and blood‐spinal cord barrier permeability following traumatic spinal cord injury. J Neurotrauma. 2014;31(6):541‐552. doi:10.1089/neu.2013.3034 24237182 PMC3949504

[5] Mun S , Han K , Hyun JK . The time sequence of gene expression changes after spinal cord injury. Cells. 2022;11(14):2236. doi:10.3390/cells11142236 35883679 PMC9324287

[6] Jin C , Zhu R , Wang Z‐W , et al. Dynamic changes in mechanical properties of the adult rat spinal cord after injury. Acta Biomater. 2023;155:436‐448. 36435440 10.1016/j.actbio.2022.11.041

[7] Moeendarbary E , Weber IP , Sheridan GK , et al. The soft mechanical signature of glial scars in the central nervous system. Nat Commun. 2017;8(1):14787. doi:10.1038/ncomms14787 28317912 PMC5364386

[8] Tran KA , DeOre BJ , Ikejiani D , et al. Matching mechanical heterogeneity of the native spinal cord augments axon infiltration in 3D‐printed scaffolds. Biomaterials. 2023;295:122061. 36842339 10.1016/j.biomaterials.2023.122061 PMC10292106

[9] Bakshi A , Fisher O , Dagci T , Himes BT , Fischer I , Lowman A . Mechanically engineered hydrogel scaffolds for axonal growth and angiogenesis after transplantation in spinal cord injury. J Neurosurg Spine. 2004;1(3):322‐329. 15478371 10.3171/spi.2004.1.3.0322

[10] Fernandez‐Zafra T , Codeluppi S , Uhlén P . An ex vivo spinal cord injury model to study ependymal cells in adult mouse tissue. Exp Cell Res. 2017;357(2):236‐242. 28587745 10.1016/j.yexcr.2017.06.002

[11] Cho J‐S , Park H‐W , Park S‐K , et al. Transplantation of mesenchymal stem cells enhances axonal outgrowth and cell survival in an organotypic spinal cord slice culture. Neurosci Lett. 2009;454(1):43‐48. 19429051 10.1016/j.neulet.2009.02.024

[12] Doussau F , Dupont J‐L , Neel D , Schneider A , Poulain B , Bossu JL . Organotypic cultures of cerebellar slices as a model to investigate demyelinating disorders. Expert Opin Drug Discovery. 2017;12(10):1011‐1022. 10.1080/17460441.2017.1356285 28712329

[13] Pandamooz S , Nabiuni M , Miyan J , Ahmadiani A , Dargahi L . Organotypic spinal cord culture: a proper platform for the functional screening. Mol Neurobiol. 2016;53:4659‐4674. 26310972 10.1007/s12035-015-9403-z

[14] Guzman‐Lenis M‐S , Navarro X , Casas C . Drug screening of neuroprotective agents on an organotypic‐based model of spinal cord excitotoxic damage. Restor Neurol Neurosci. 2009;27(4):335‐349. 19738326 10.3233/RNN-2009-0482

[15] Gerardo‐Nava J , Mayorenko II , Grehl T , Steinbusch HW , Weis J , Brook GA . Differential pattern of neuroprotection in lumbar, cervical and thoracic spinal cord segments in an organotypic rat model of glutamate‐induced excitotoxicity. J Chem Neuroanat. 2013;53:11‐17. 24126226 10.1016/j.jchemneu.2013.09.007

[16] Patar A , Dockery P , Howard L , McMahon SS . Cell viability in three ex vivo rat models of spinal cord injury. J Anat. 2019;234(2):244‐251. 30417349 10.1111/joa.12909 PMC6326831

[17] Moser KV , Schmidt‐Kastner R , Hinterhuber H , Humpel C . Brain capillaries and cholinergic neurons persist in organotypic brain slices in the absence of blood flow. Eur J Neurosci. 2003;18(1):85‐94. 12859340 10.1046/j.1460-9568.2003.02728.x

[18] Zehendner CM , Wedler HE , Luhmann HJ . A novel in vitro model to study pericytes in the neurovascular unit of the developing cortex. PLoS One. 2013;8(11):e81637. 24278454 10.1371/journal.pone.0081637 PMC3836850

[19] Senarathna J , Rege A , Li N , Thakor NV . Laser speckle contrast imaging: theory, instrumentation and applications. IEEE Rev Biomed Eng. 2013;6:99‐110. 23372086 10.1109/RBME.2013.2243140

[20] Lesage F , Brieu N , Dubeau S , Beaumont E . Optical imaging of vascular and metabolic responses in the lumbar spinal cord after T10 transection in rats. Neurosci Lett. 2009;454(1):105‐109. 19429064 10.1016/j.neulet.2009.02.060

[21] Khaing ZZ , Cates LN , Hyde JE , Hammond R , Bruce M , Hofstetter CP . Transcutaneous contrast‐enhanced ultrasound imaging of the posttraumatic spinal cord. Spinal Cord. 2020;58(6):695‐704. 31965060 10.1038/s41393-020-0415-9

[22] Khaing ZZ , Cates LN , Hyde J , et al. Contrast‐enhanced ultrasound for assessment of local hemodynamic changes following a rodent contusion spinal cord injury. Mil Med. 2020;185(Suppl_1):470‐475. 32074323 10.1093/milmed/usz296

[23] Koser DE , Moeendarbary E , Hanne J , Kuerten S , Franze K . CNS cell distribution and axon orientation determine local spinal cord mechanical properties. Biophys J. 2015;108(9):2137‐2147. 25954872 10.1016/j.bpj.2015.03.039 PMC4423070

[24] Mitra P , Brownstone RM . An in vitro spinal cord slice preparation for recording from lumbar motoneurons of the adult mouse. J Neurophysiol. 2012;107(2):728‐741. 22031766 10.1152/jn.00558.2011

[25] Sipski ML , Jackson AB , Gomez‐Marin O , Estores I , Stein A . Effects of gender on neurologic and functional recovery after spinal cord injury. Arch Phys Med Rehabil. 2004;85(11):1826‐1836. doi:10.1016/j.apmr.2004.04.031 15520978

[26] Liao Y , Smyth GK , Shi W . The R package Rsubread is easier, faster, cheaper and better for alignment and quantification of RNA sequencing reads. Nucleic Acids Res. 2019;47(8):e47. doi:10.1093/nar/gkz114 30783653 PMC6486549

[27] Love MI , Huber W , Anders S . Moderated estimation of fold change and dispersion for RNA‐seq data with DESeq2. Genome Biol. 2014;15(12):550. doi:10.1186/s13059-014-0550-8 25516281 PMC4302049

[28] Durinck S , Spellman PT , Birney E , Huber W . Mapping identifiers for the integration of genomic datasets with the R/Bioconductor package biomaRt. Nat Protoc. 2009;4(8):1184‐1191. doi:10.1038/nprot.2009.97 19617889 PMC3159387

[29] Subramanian A , Tamayo P , Mootha VK , et al. Gene set enrichment analysis: a knowledge‐based approach for interpreting genome‐wide expression profiles. Proc Natl Acad Sci U S A. 2005;102(43):15545‐15550. doi:10.1073/pnas.0506580102 16199517 PMC1239896

[30] Khaing ZZ , Cates LN , DeWees DM , et al. Contrast‐enhanced ultrasound to visualize hemodynamic changes after rodent spinal cord injury. J Neurosurg Spine. 2018;29(3):306‐313. doi:10.3171/2018.1.SPINE171202 29905521

[31] Partyka PP , Godsey GA , Galie JR , et al. Mechanical stress regulates transport in a compliant 3D model of the blood–brain barrier. Biomaterials. 2017;115:30‐39. 27886553 10.1016/j.biomaterials.2016.11.012

[32] Carmel JB , Galante A , Soteropoulos P , et al. Gene expression profiling of acute spinal cord injury reveals spreading inflammatory signals and neuron loss. Physiol Genomics. 2001;7(2):201‐213. doi:10.1152/physiolgenomics.00074.2001 11773606

[33] Arndt SO , Vogt AB , Markovic‐Plese S , et al. Functional HLA‐DM on the surface of B cells and immature dendritic cells. EMBO J. 2000;19(6):1241‐1251. doi:10.1093/emboj/19.6.1241 10716924 PMC305665

[34] Casasola‐LaMacchia A , Ritorto MS , Seward RJ , et al. Human leukocyte antigen class II quantification by targeted mass spectrometry in dendritic‐like cell lines and monocyte‐derived dendritic cells. Sci Rep. 2021;11(1):1028. doi:10.1038/s41598-020-77024-y 33441579 PMC7807004

[35] Lenormand C , Bausinger H , Gross F , et al. HLA‐DQA2 and HLA‐DQB2 genes are specifically expressed in human Langerhans cells and encode a new HLA class II molecule. J Immunol. 2012;188(8):3903‐3911. doi:10.4049/jimmunol.1103048 22407913

[36] Choo AM , Liu J , Lam CK , Dvorak M , Tetzlaff W , Oxland TR . Contusion, dislocation, and distraction: primary hemorrhage and membrane permeability in distinct mechanisms of spinal cord injury. J Neurosurg Spine. 2007;6(3):255‐266. 17355025 10.3171/spi.2007.6.3.255

[37] Leal M , Reid S , McCreedy D . L‐selectin shedding reduces neutrophil accumulation AND improves long‐term recovery after spinal cord injury. J Neurotrauma. 2022;40(15‐16):21.

[38] Harmon JN , Khaing ZZ , Hyde JE , Hofstetter CP , Tremblay‐Darveau C , Bruce MF . Quantitative tissue perfusion imaging using nonlinear ultrasound localization microscopy. Sci Rep. 2022;12(1):21943. doi:10.1038/s41598-022-24986-w 36536012 PMC9763240

[39] Horn EM , Theodore N , Assina R , Spetzler RF , Sonntag VK , Preul MC . The effects of intrathecal hypotension on tissue perfusion and pathophysiological outcome after acute spinal cord injury. Neurosurg Focus. 2008;25(5):E12. doi:10.3171/FOC.2008.25.11.E12 18980472

[40] Paone LS , Szkolnicki M , DeOre BJ , et al. Effects of drag‐reducing polymers on hemodynamics and whole blood‐endothelial interactions in 3D‐printed vascular topologies. ACS Appl Mater Interfaces. 2024;16(12):14457‐14466. doi:10.1021/acsami.3c17099 38488736 PMC10982934

[41] Lam CJ , Assinck P , Liu J , Tetzlaff W , Oxland TR . Impact depth and the interaction with impact speed affect the severity of contusion spinal cord injury in rats. J Neurotrauma. 2014;31(24):1985‐1997. doi:10.1089/neu.2014.3392 24945364 PMC4245874

[42] Donnelly DJ , Popovich PG . Inflammation and its role in neuroprotection, axonal regeneration and functional recovery after spinal cord injury. Exp Neurol. 2008;209(2):378‐388. 17662717 10.1016/j.expneurol.2007.06.009 PMC2692462

[43] Popovich PG , Hickey WF . Bone marrow chimeric rats reveal the unique distribution of resident and recruited macrophages in the contused rat spinal cord. J Neuropathol Exp Neurol. 2001;60(7):676‐685. 11444796 10.1093/jnen/60.7.676

[44] Nimmerjahn A , Kirchhoff F , Helmchen F . Resting microglial cells are highly dynamic surveillants of brain parenchyma in vivo. Science. 2005;308(5726):1314‐1318. 15831717 10.1126/science.1110647

[45] Davalos D , Grutzendler J , Yang G , et al. ATP mediates rapid microglial response to local brain injury in vivo. Nat Neurosci. 2005;8(6):752‐758. 15895084 10.1038/nn1472

[46] Popovich PG , Streit WJ , Stokes BT . Differential expression of MHC class II antigen in the contused rat spinal cord. J Neurotrauma. 1993;10(1):37‐46. doi:10.1089/neu.1993.10.37 8320731

[47] Tome D , Dias MS , Correia J , Almeida RD . Fibroblast growth factor signaling in axons: from development to disease. Cell Commun Signal. 2023;21(1):290. doi:10.1186/s12964-023-01284-0 37845690 PMC10577959

