# Supplementary material for: An ex vivo injury model perfused with whole blood reveals rapid mechanical and transcriptional changes within the spinal cord
Source: Bioeng Transl Med. 2026 Mar 23;11(4):e70139. doi: 10.1002/btm2.70139 (PMC13327618; doi:10.1002/btm2.70139)
Supplement: Supplementary file 1 — Figure S1. Photograph of a spinal cord section prior to indentation by the mechanical testing system. Figure S2. Schematic showing the quantification method of (A) a representative IHC image for (B) Evans blue ratio. Figure S3. Mechanical testing analysis comparing perfused versus non‐perfused samples. Percentage decrease in mechanical properties from the time of injury to the second measurement in cords. Figure S4. Expression of cell type‐specific markers for (A) astrocytes, (B) microglia, (C) neurons, and (D) oligodendrocytes. Figure S5. Volcano plot comparing differential gene expression between cords processed at 15 and 60 min post‐injury. [file BTM2-11-e70139-s001.docx]

SUPPLEMENTAL FIGURES


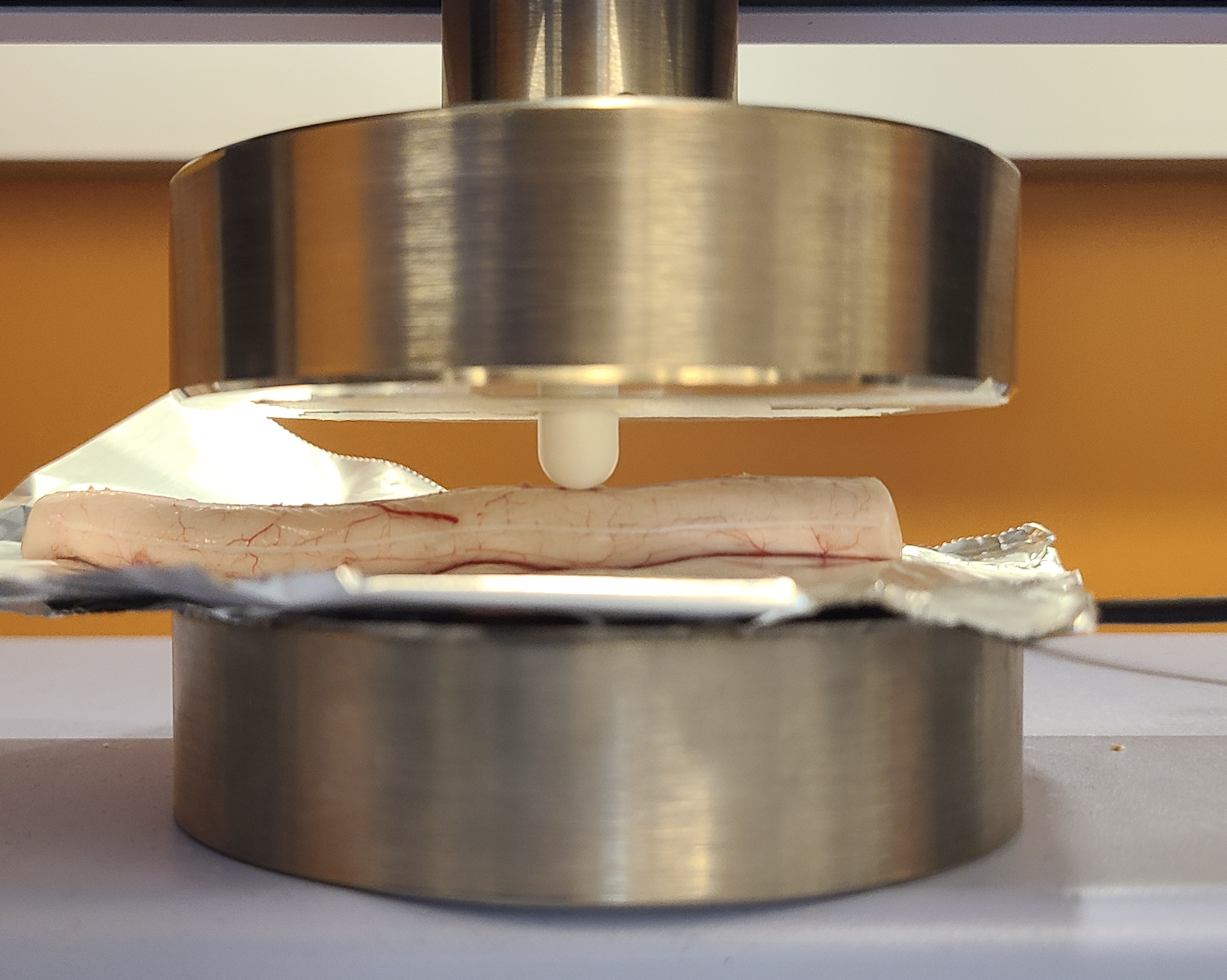


Supplemental Figure 1: Photograph of a spinal cord section prior to indentation by the mechanical testing system.


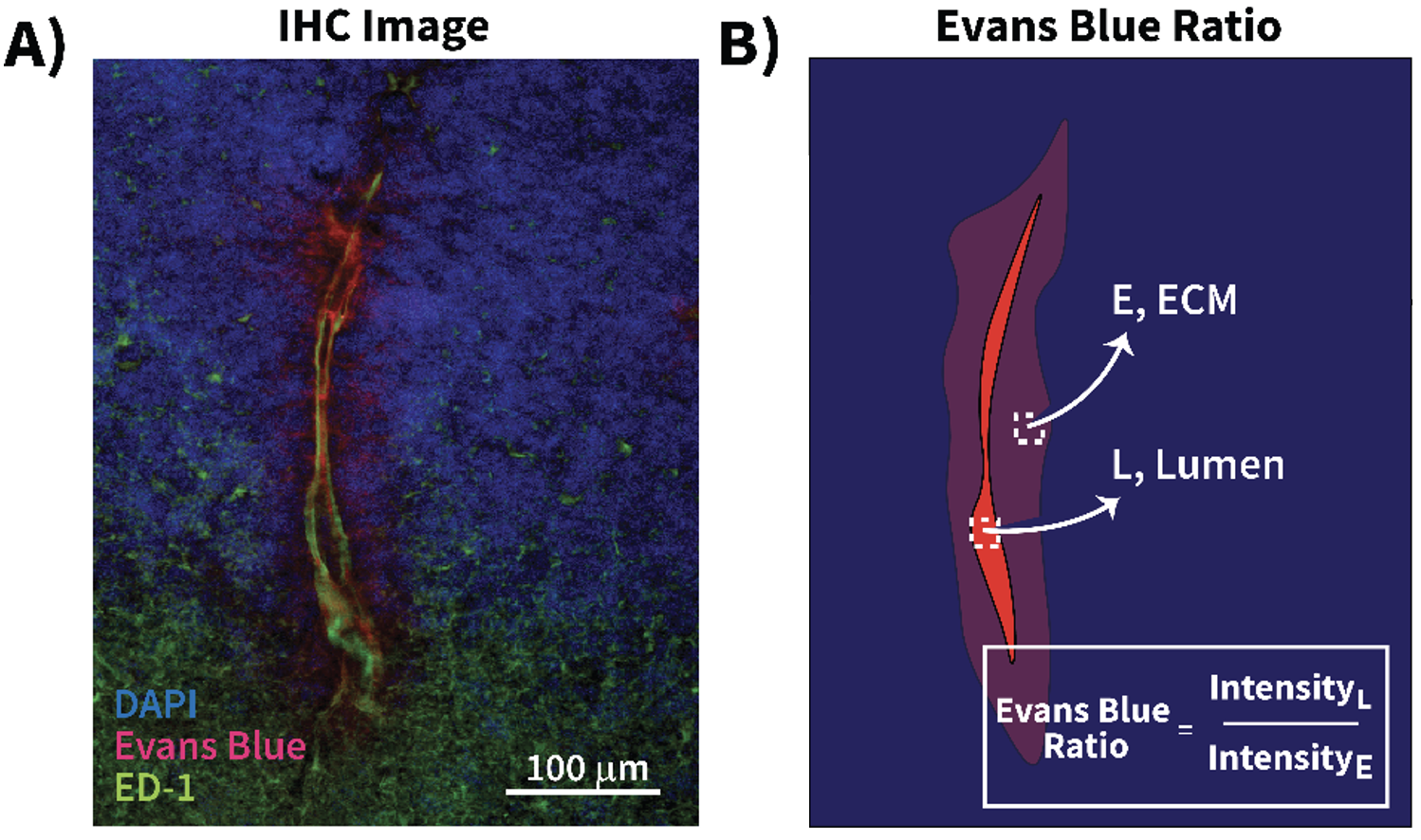


Supplemental Figure 2: Schematic showing the quantification method of (A) a representative IHC image for (B) Evans blue ratio.


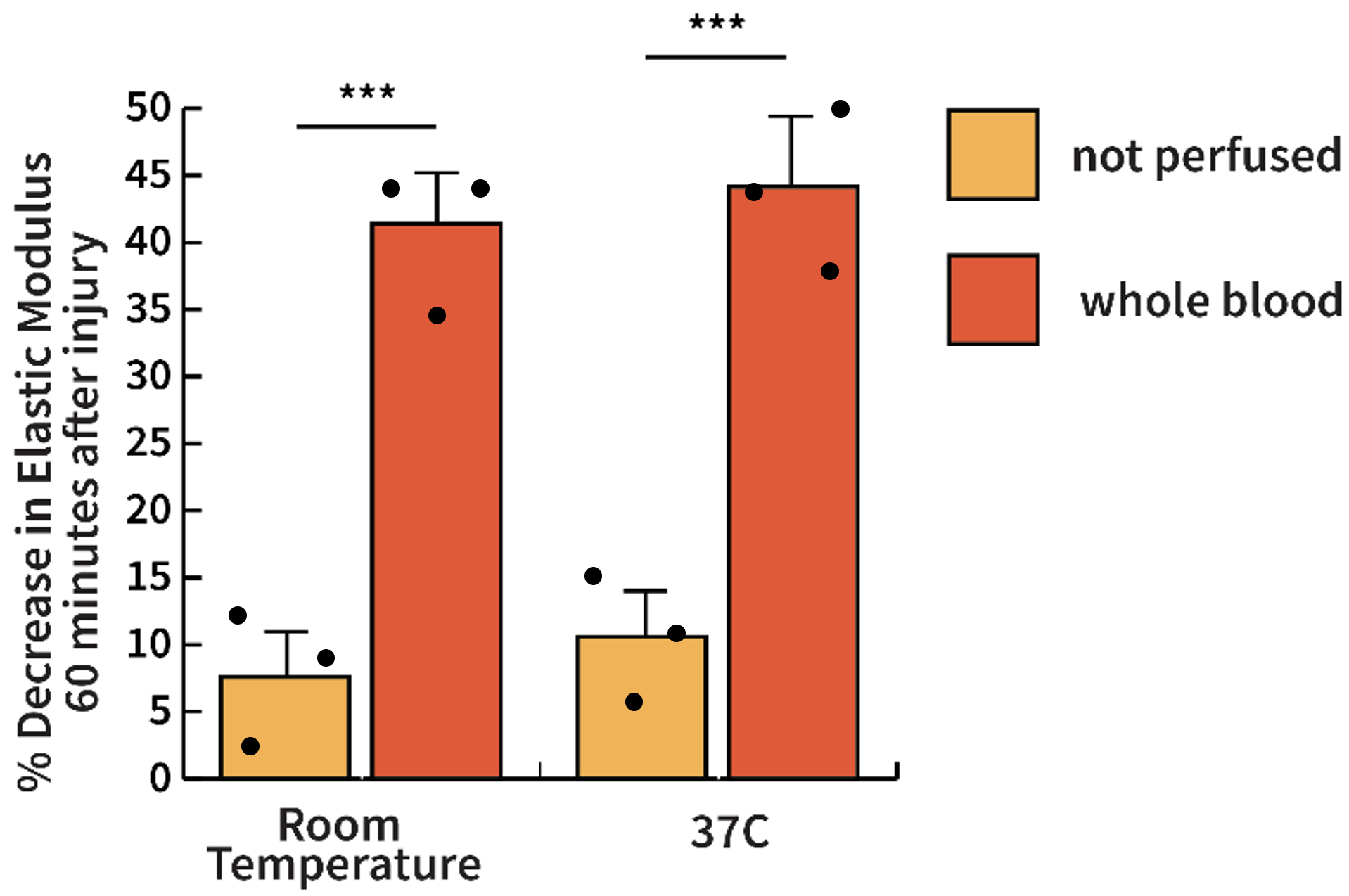


Supplemental Figure 3: Mechanical testing analysis comparing perfused vs. non-perfused samples. Percentage decrease in mechanical properties from the time of injury to the second measurement in cords.


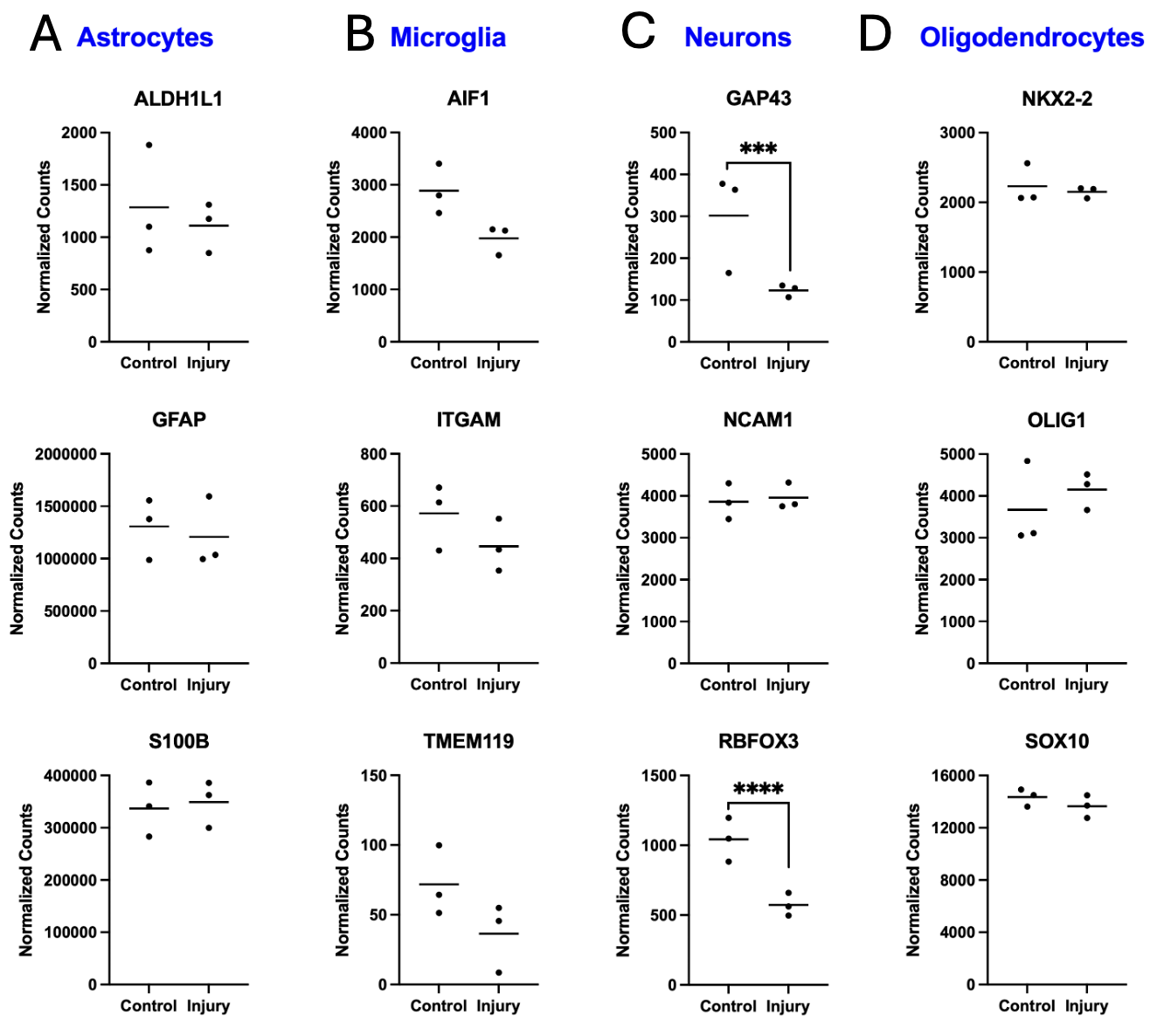


Supplemental Figure 4: Expression of cell type-specific markers for (A) astrocytes, (B) microglia, (C) neurons, and (D) oligodendrocytes.


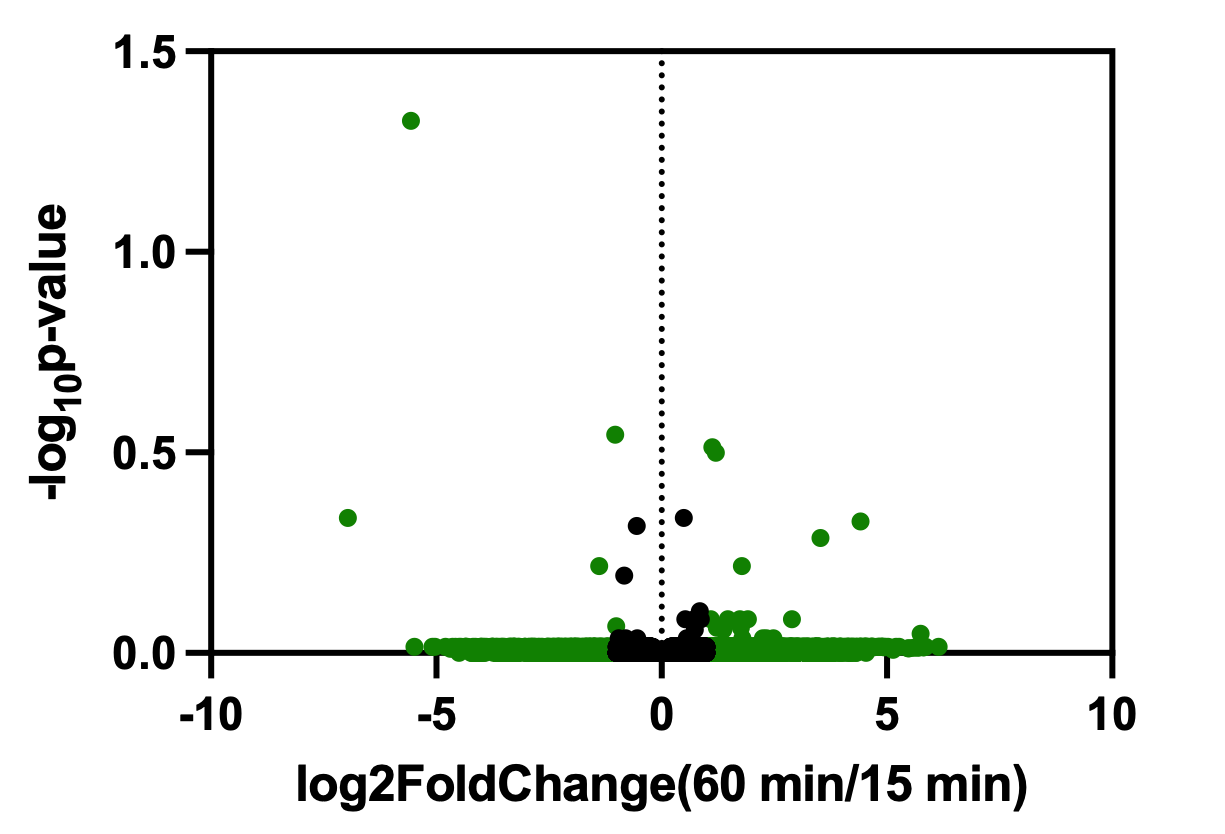


Supplemental Figure 5: Volcano plot comparing differential gene expression between cords processed at 15 and 60 minutes post-injury.
